# Elucidating the importance and regulation of key enhancers for human *MEIS1* expression

**DOI:** 10.1038/s41375-022-01602-4

**Published:** 2022-05-27

**Authors:** Ping Xiang, Xining Yang, Leo Escano, Ishpreet Dhillon, Edith Schneider, Jack Clemans-Gibbon, Wei Wei, Jasper Wong, Simon Xufeng Wang, Derek Tam, Yu Deng, Eric Yung, Gregg B. Morin, Pamela A. Hoodless, Martin Hirst, Aly Karsan, Florian Kuchenbauer, R. Keith Humphries, Arefeh Rouhi

**Affiliations:** 1 Terry Fox Laboratory, British Columbia Cancer Research Centre, Vancouver, Canada; 2 Department of Medical Genetics, University of British Columbia, Vancouver, Canada; 3 Canada’s Michael Smith Genome Sciences Centre, British Columbia Cancer Research Centre, Vancouver, Canada; 4 Department of Pathology and Laboratory Medicine, University of British Columbia, Vancouver, Canada; 5 School of Biomedical Engineering, University of British Columbia, Vancouver, Canada; 6 Leukemia/Bone Marrow Transplant Program of British Columbia, Vancouver General Hospital, BC Cancer, Vancouver, Canada; 7 Department of Medicine, University of British Columbia, Vancouver, Canada

**Keywords:** Cancer epigenetics, Cancer epigenetics

## Abstract

Myeloid ecotropic virus insertion site 1 (MEIS1) is essential for normal hematopoiesis and is a critical factor in the pathogenesis of a large subset of acute myeloid leukemia (AML). Despite the clinical relevance of MEIS1, its regulation is largely unknown. To understand the transcriptional regulatory mechanisms contributing to human MEIS1 expression, we created a knock-in green florescent protein (GFP) reporter system at the endogenous *MEIS1* locus in a human AML cell line. Using this model, we have delineated and dissected a critical enhancer region of the *MEIS1* locus for transcription factor (TF) binding through in silico prediction in combination with oligo pull-down, mass-spectrometry and knockout analysis leading to the identification of FLI1, an E-twenty-six (ETS) transcription factor, as an important regulator of *MEIS1* transcription. We further show direct binding of FLI1 to the *MEIS1* locus in human AML cell lines as well as enrichment of histone acetylation in MEIS1-high healthy and leukemic cells. We also observe a positive correlation between high *FLI1* transcript levels and worse overall survival in AML patients. Our study expands the role of ETS factors in AML and our model constitutes a feasible tool for a more detailed understanding of transcriptional regulatory elements and their interactome.

## Introduction

Myeloid ecotropic virus insertion site 1 (MEIS1) is a HOX co-factor known to be necessary for normal hematopoiesis [1, 2] and it is implicated in a wide range of leukemias due to its deregulated overexpression [3]. Previous work, including that of our own, has shown that MEIS1 acts as an important driver for leukemogenesis [3]. MEIS1 is dysregulated in a large subset of acute myeloid leukemia (AML) patients [4, 5] through as yet undefined mechanisms and is critical for the maintenance of leukemia stem cells [6–9]. Decreasing *MEIS1* expression via shRNA mediated knockdown has been shown to significantly reduce leukemic stem cell potential [7]. Identifying the transcriptional regulators of *MEIS1* with the long-term goal of identifying ways to alter *MEIS1* expression could be beneficial for developing new therapies in MEIS1-dependent leukemias [4].

The *MEIS1* locus spans some 175 kb of the genome and recent findings suggest that this large genomic region encompasses multiple regulatory regions including several enhancers. Two such studies have identified candidate cis-regulatory regions based on sequence conservation and with a focus on regulation of *MEIS1* in early development [10, 11]. In our previous work we exploited several human leukemic cells lines with variable levels of *MEIS1* expression and identified three candidate enhancer regions based on epigenetic markers [12]: enhancer region 1 (E1) at −2kb upstream; enhancer region 2 (E2) at +10.6 kb downstream, inside intron 6; and Enhancer region 3 (E3) at +140 kb downstream of the transcriptional start site. While E1 displayed an active chromatin status in all the *MEIS1* expressing cells, an active E2 region was more associated with high *MEIS1* expressing cell lines and an active E3 region was more associated with medium level *MEIS1* expressing cell lines [12]. Using chromosome conformation capture (3 C) assay, we also showed that these three enhancer regions interact with the promoter region of *MEIS1*. A recent report by Lin et al. indicates the region encompassing E1 and E2 as a super-enhancer in Ewing sarcoma with high H3K27Ac correlating to high *MEIS1* expression [13]. In the current study, we have utilized CRISPR-Cas9 genome editing [14, 15] to further characterize these enhancers in human AML cells as well as identify the key transcription factors (TFs) associated with their function.

## Materials and methods

### Generation of tagged U937 cell lines

U937 cells were obtained from the American Tissue Culture Collection (ATCC). U937 cell cultures were maintained at 37 °C in 5% CO_2_ in RPMI 1640 supplemented with 10% Performance Plus FBS and Pen/Strep (RPMI++) (Gibco, Thermo Fishier Scientific, Waltham, MA, USA) and frequently mycoplasma tested. A GFP-P2A-HA encoding sequence was tagged to the start codon (ATG) of MEIS1 gene as previously published [16], except that the donor template for homologous recombination was constructed containing the sequence on each side of the ATG of human *MEIS1* (chr2: 66,662,989-66,662,991, hg19). Generation and selection of cells are further described in the Supplementary Methods and Material section.

### Flow cytometry analysis and FACS

Cells were analyzed or sorted using a BD Fortessa cell analyzer or Aria Fusion (BD Biosciences, San Jose, CA, USA) respectively. Data acquisition was performed in the presence of 1 μM DAPI for the gating of viable cells. Data analysis was performed using the FlowJo software (TreeStar, Ashland, OR, USA).

### Reverse transcription and real-time PCR

Total RNA was isolated using Trizol (Thermo Fishier Scientific, Waltham, MA, USA) following the manufacturer’s instructions. cDNA synthesis and Real-time PCR were performed as described previously [12]. Primer sequences are provided in the Supplementary Methods and Material section.

### Western blotting

Cells were lysed in RIPA buffer subjected to 5-12% SDS-PAGE, and transferred to a nitrocellulose membrane (Thermo Fishier Scientific, Waltham, MA, USA). Proteins extracted from ~0.2 million cells were loaded in each lane. The blot was incubated with a primary anti-HA rabbit monoclonal antibody (C29F4, New England Biolab, Ipswich, Massachusetts, USA) or an anti-ERG/FLI1 rabbit monoclonal antibody (ab92513, Abcam, Cambridge, United Kingdom). An anti-ACTIN monoclonal antibody (G043, Abm, Vancouver, BC, Canada) was used as control. Blots were visualized by ECL (GE Healthcare, Little Chalfont, United Kingdom).

### CRISPR-Cas9 lentiviral vector

The CRISPR-Cas9 lentiviral vector used in this study was generated by substituting the Puro sequence with Cherry from CRISPR-Cas9 vector designed in Feng Zhang’s group (Addgene Plasmid 49535, Cambridge, Massachusetts, USA). Cloning was done accordingly to their recommended protocol (https://media.addgene.org/data/plasmids/52/52961/52961-attachment_B3xTwla0bkYD.pdf).

### PCR amplification and sequencing of the targeted region

A 500 bp region surrounding the predicted Cas9 cutting sites was amplified from the sorted Cherry^+^ cells after the lentiCRISPR virus transformation (Supplementary Methods and Material section). Sequencing was performed using Illumina MiSeq. Reads were aligned to their respective reference amplicons and editing efficiency was quantified by CRISPResso v.1.0.12 [17]. High quality reads (phred >30) were filtered and paired before alignment. Substitutions, insertions, and deletions within 5 bp of the gRNA-predicted cutting sites were considered as non-homologous end joining (NHEJ) events.

### Chromosome conformation capture (3C) assay

3C assay was performed as previously described [12, 18]. To capture the interaction between the tagged *MEIS1* promoter and candidate enhancer regions, the cut site for the restriction enzyme, BsrG I, located in the GFP gene of the tagged allele, was used to generate the fragments for subsequent ligation. Primers used are listed in the Supplementary Methods and Material section.

### DNA pull-down assay

DNA pull-down was performed using the protocol by Andrews et al. [19] with some modifications. Instead of the Streptavidin-agarose beads, dynabeads M280 (Thermo Fishier Scientific, Waltham, MA, USA) were used according to the manufacturer suggested protocol.

### Chromatin immunoprecipitation (ChIP) assay

ChIP assays on U937 cells were performed as previously described with modifications [20] detailed in the Supplementary Methods and Material section. Chromatin immunoprecipitation followed by sequencing (ChIP-seq) and analysis of mouse Hoxa9/Meis1 and Hoxa9/∆HDMeis1 cells as well as the NUP98-HOXD13/Meis1 and NUP98-HOXA10 homeodomain/Meis1 were performed as previously described [21].

### Statistical analysis

Welch’s two-tailed t-test was used to calculate p-values. Statistical analysis was performed using the GraphPad Prism 8.0 (GraphPad Software, Inc., San Diego, CA).

### Survival analysis

Primary AML patient RNA sequencing data was obtained from the Beat AML trial (NCT03013998) [22]. Both bone marrow and peripheral blood patient samples corresponding to time of initial diagnosis were selected for downstream analysis. Raw, aligned counts were then normalized using edgeR [23, 24]. To assess the impact of gene expression on patient survival, the R package Maximally Selected Rank Statistics [25] was used to categorize patients into high and low expressing groups. To determine significant differences in survival, log rank tests were done using the R package survival [26] and then visualized using survminer [27].

## Results

### Tagging endogenous MEIS1 in the U937 human AML cell line

**Fig. 1:**
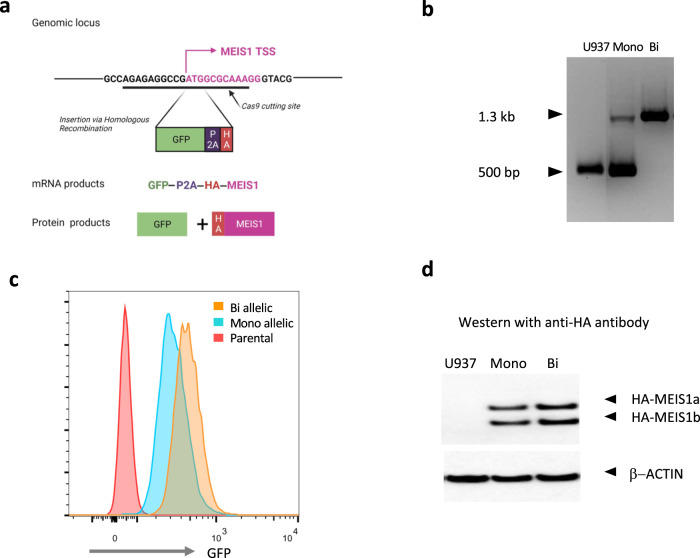
Tagging endogenous *MEIS1* in U937 human AML cell line. **a** Introducing a GFP reporter, a P2A self-cleaving peptide tag and an HA tag at the translation start site of *MEIS1* in U937 cells. **b** Confirmation of the insertion of the tag into the correct genomic locus at both alleles (bi) or one allele (mono) in independent single clones by PCR. **c** GFP levels of tagged clones measured by flow cytometry. **d** HA-tagged MEIS1 protein levels measured by western blot analysis using an anti-HA tagged antibody.

We first established an efficient method to track MEIS1 expression levels by introducing a GFP reporter, a P2A self-cleaving peptide tag and a hemagglutinin (HA) tag at its translation start site as previously described [16] in U937 cells, a MEIS1 high expressing human AML cell line [12] (Fig. 1a). This reporter construct allows the co-transcription of *GFP* and HA-tagged *MEIS1* using the endogenous *MEIS1* transcriptional regulatory machinery. The self-cleaving action of the P2A sequence post co-translation, leads to the simultaneous generation of GFP and HA-tagged MEIS1 proteins. The insertion of the tag into the correct genomic locus at both alleles (bi) or one allele (mono) in independent single clones was confirmed by PCR analysis (Fig. 1b). Flow cytometry for GFP levels (Fig. 1c) and western blot analysis using an anti-HA tag antibody (Fig. 1d) showed higher GFP expression and higher HA tagged protein levels in the biallelic clone. These results were consistent with the higher tagged *MEIS1* mRNA levels detected in the biallelic clone as measured by primers spanning the tag region to exon 2 of *MEIS1* (Supplementary Fig. S1a, left panel). For the mono-allelic tagged clones, we noticed that editing still occurred in the second allele at the beginning of the open reading frame of *MEIS1*, resulting in a frame shift mutation leading to the abrogation of *MEIS1* expression from the wildtype allele (Supplementary Fig. S1b). Subsequently, these mono-allelic tagged clones expressed about half the amount of total *MEIS1* transcript compared to the parental or the biallelic tagged clones (Supplementary Fig. S1a, right panel).

### CRISPR-Cas9 mediated targeting of candidate enhancer regions

**Fig. 2:**
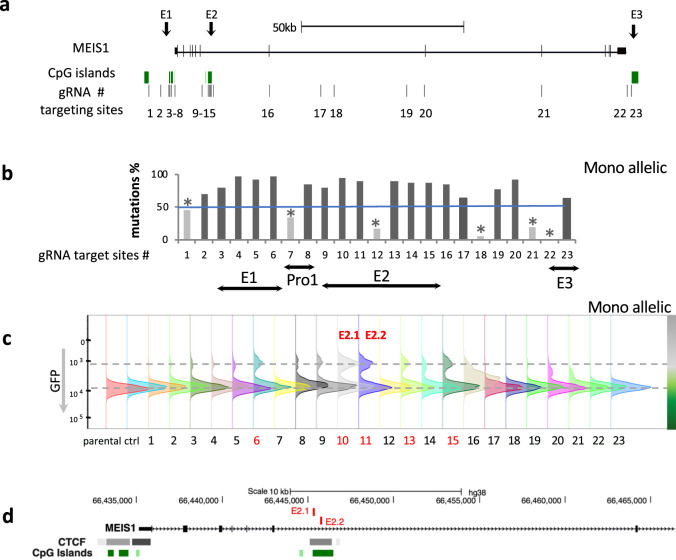
CRISPR-Cas9 mediated targeting of candidate enhancer regions. **a** Introducing random mutations (Indels) at the *MEIS1* enhancer regions in U937 cells. #1-23 denote sites selected for gRNA mediated genome editing. **b** The editing frequency of each gRNA in the bulk population from a mono-allelic targeted clone measured by MiSeq analysis. **c** GFP levels of the gRNA targeted regions measured by flow cytometry. Regions targeted by gRNAs #10 and #11 are referred to as E2.1 and E2.2 respectively. **d** HG38 UCSC genome browser depiction of E2.1 and E2.2 targeted region. gRNA: guide RNA.

To identify the key sequences important to the function of the *MEIS1* enhancer regions, we introduced random mutations (Indels) at the E1, E2 and E3 regions, as well as a few along the gene body based on our previously published H3 acetylation and DNaseI hypersensitivity peaks in U937 [12] denoting open chromatin (Fig. 2a). For this purpose, we utilized the Cherry colored lentiviral CRISPR-Cas9 mediated genome editing vector system (Supplementary Fig. S2) in the mono-allelic MEIS1-GFP-tagged U937 cells. 23 sites within the *MEIS1* locus were selected for guide RNA (gRNA) mediated genome editing (Supplementary Table 1) with a focus on the most active enhancer regions, E1 and E2 (Fig. 2a). We first evaluated the editing frequency of each gRNA in transduced cells through MiSeq analysis of the targeted genomic regions in the bulk population from a mono-allelic targeted clone. Of the 23 targeted sites, 17 gRNAs yielded more than 50% mutation frequency around the predicted gRNA-targeted Cas9 cutting site whereas six had less than 50% editing efficiency in the bulk mono-allelic cells and therefore could not be evaluated (Fig. 2b). Targeting was associated with a decrease in the proportion of GFP^+^ cells (Fig. 2c and Supplementary Table 1). These targeted regions included a site within the distal promoter region of *MEIS1*(#6) and 4 sites (#10, #11, #13 and #15) within the CpG island of intron 6 which correspond to the previously identified E2 region (Fig. 2c). Regions targeted by gRNAs #10 and #11 yielded the highest proportion of GFP^−^ cells (Fig. 2c and Supplementary Table 1). These two gRNAs, hereafter referred to as E2.1 (gRNA #10) and E2.2 (gRNA #11) are 395 bp apart, with varied indel size distribution (Supplementary Fig. 2), and target a region which has been shown to be bound by the insulator related transcription factor (TF) CTCF in several cell lines (Fig. 2d) included in the ENCODE project [12]. We therefore, further focused on the E2 region, specifically sites affected by the E2.1 and E2.2 gRNAs, which displayed the strongest negative effect on *MEIS1* transcription when targeted in the mono-allelically (Fig. 2c and Supplementary Table 1) as well as the biallelically tagged cells (Supplementary Table 1).

### Characterization of the E2 enhancer region

**Fig. 3:**
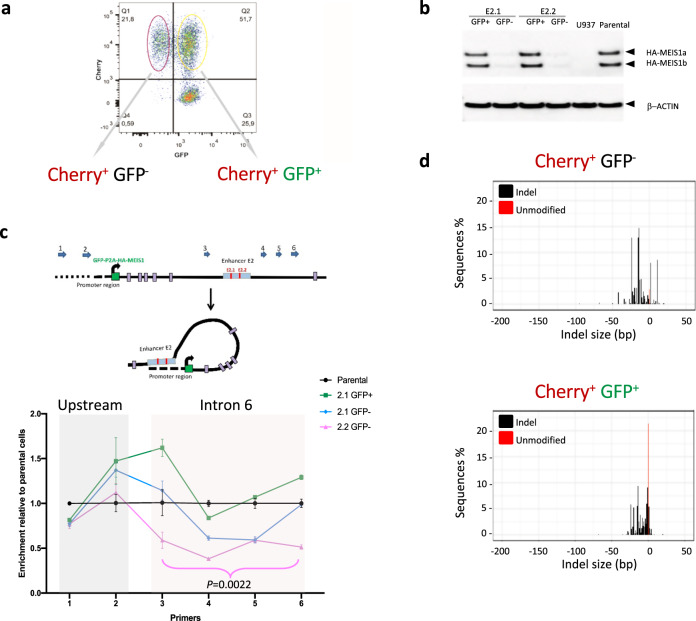
Characterization of the E2 enhancer region. **a** Sorting the GFP^−^ and GFP^+^ populations from the E2.1 and E2.2 gRNA targeted Cherry^+^ mono-allelic MEIS1-GFP-tagged cells. **b** MEIS1 protein level of Cherry^+^GFP^+^ cells and Cherry^+^GFP^−^ cells from E2.1 and E2.2 targeted cells measured by western blot analysis. **c** Upper panel: schematic depiction of interactions between the promoter (curved black arrow depicts transcriptional start site and direction of transcription) and the E2 region. Lower panel: chromosome conformation capture (3C) assay measuring this interaction in mono-allelic MEIS1-GFP-tagged Cherry^+^GFP^+^ cells and Cherry^+^GFP^−^ cells from E2.1 and E2.2 targeted GFP^−^ cells, compared to the parental cells. Welch’s two-tailed t-test was used to calculate *p* values. **d** Targeted site of the E2.2 region in MEIS1-GFP-tagged Cherry^+^GFP^+^ cells and Cherry^+^GFP^−^ cells detected by sequencing. The height of the red bars show fraction of non-targeted/unmodified gDNA.

To analyze the effect of genome editing at the E2 enhancer region on *MEIS1* expression, we sorted the GFP^−^ and GFP^+^ populations (Supplementary Fig. S2a) from the E2.1 and E2.2 gRNA targeted Cherry^+^ mono-allelic MEIS1-GFP-tagged cells after genome editing at the E2.1 and E2.2 sites (Fig. 3a and Supplementary Fig. S2a). For both regions, compared to the GFP^+^ cells, GFP^−^ cells exhibited a decreased tagged *MEIS1* expression at both the mRNA (Supplementary Fig. S2b) and protein levels (Fig. 3b). Using chromosome conformation capture (3C) assay, we had previously shown that the E2 region forms a strong interaction with the *MEIS1* promoter via looping [12]. To gauge the effect of genome editing at the E2.1 and E2.2 sites on promoter/enhancer interaction, we performed 3C assay on the mono-allelic MEIS1-GFP-tagged Cherry^+^/GFP^−^ sorted E2.1 and E2.2 targeted cells. As controls we also performed 3C assay on the mono-allelic MEIS1-GFP-tagged parental (untargeted) and MEIS1-GFP-tagged Cherry^+^/GFP^+^ sorted cells. We detected a significantly (*p* = 0.0022) decreased interaction between the promoter and the intron 6 region surrounding the E2 region in E2.2 targeted cells compared to the parental cells in four tested sub-regions [3–6] (Fig. 3c). This decreased interaction was specific to intron 6 since the contact interaction of the upstream regions [1, 2] remained unchanged in gRNA targeted cells compared to parental cells (Fig. 3c). To further narrow down the critical region essential to *MEIS1* expression targeted by E2.1 and E2.2, we PCR amplified and sequenced the E2 region in GFP^+^ and GFP^−^ cells (Supplementary Fig. S2c). For both the E2.1 and E2.2 targeted sites, compared to the GFP^−^ population, there was more unmodified sequence within the GFP^+^ population and the mutations are more centered on the predicted Cas9 cutting site. Consistent with the sequence analysis from the bulk population, the indels are smaller and more clustered for the E2.2 gRNA targeted site (majority within 25 bp from the predicted cutting site) compared to the E2.1 gRNA targeted site where indels span a comparatively larger genomic range for either the GFP^−^ or GFP^+^ populations (Fig. 3d and Supplementary Fig. S2d and Supplementary Fig. S3). These results are consistent with the interpretation that the DNA sequence within the E2.2 gRNA targeting site is highly critical to this region’s enhancer function, while the enhancer function is further influenced by the larger genomic region surrounding the E2.1 gRNA target site (Supplementary Fig. S3). We further confirmed the relevance of the E2.2 region to chromatin interaction and structure of the *MEIS1* locus by Hi-C analysis in parental U937 (MEIS1-GFP-tagged Cherry^+^GFP^+^) and E2.2 CRISPR’d (MEIS1-GFP-tagged Cherry^+^GFP^−^) cells (Supplementary Fig. S4a, b) where we observed a reduction in chromatin interactions and a shift to a closed chromatin state in the E2.2 mutated GFP^−^ cells.

### Identifying TFs associated with E2.2 gRNA targeting site

**Fig. 4:**
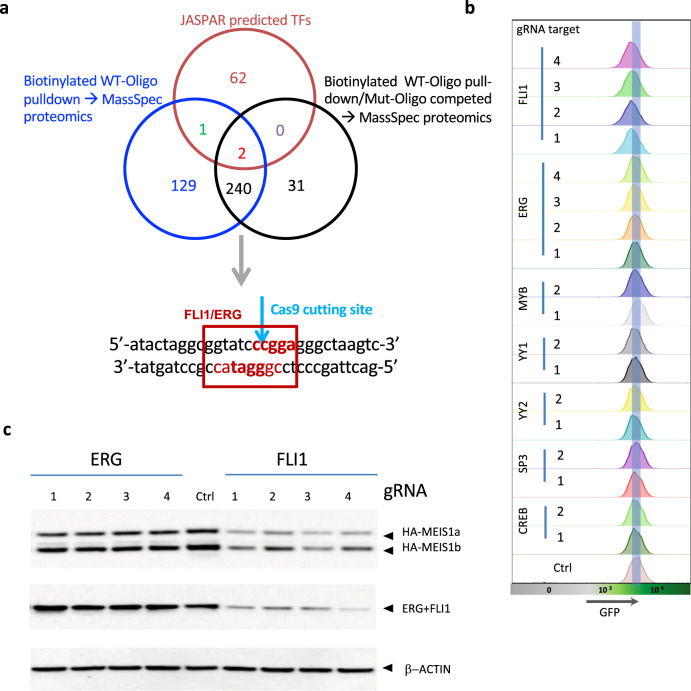
FLI1 is a predicted transcription factor of *MEIS1*. **a** Identified transcription factors via Jasper analysis and mass-spectrometry with biotinylated wildtype (WT)-oligo pull-down with and without mutant (Mut)-oligo competition. **b** Targeting each predicted transcription factor via CRISPR-Cas9 in MEIS1-GFP-tagged U937 cells and measuring GFP levels by flow cytometry. The shaded horizontal bar delineates the histogram GFP-peak of the parental cell line (**c**) HA-tagged MEIS1 protein levels in ERG and FLI1 CRISPR-targeted cells measured by western blot analysis. Four specific sgRNA [1–4] were used for targeting FLI1 and ERG.

To identify the TFs binding to the E2 region, we further scrutinized the E2.2 indel region for loss of TF binding sites. We hypothesized that binding sites of some critical regulators of *MEIS1* should be within the E2.2 gRNA targeting site which made it feasible to identify these TFs through sequence analysis and in vitro oligo pull-down. We first performed TF prediction analysis around the E2.2 gRNA targeted site via JASPAR (https://jaspar.genereg.net/) (Supplementary Table S2). In addition, we designed a biotinylated oligo (WT) for this site and performed a protein pull-down experiment followed by mass spectrometry analysis to identify the TF candidates (Supplementary Table S2). Additionally, we used a non-biotinylated mutated (Mut) oligo (mutate at the Cas9 cutting site) to compete with the wildtype oligo in another protein pull-down followed by mass spectrometry analysis. The overlap between the Jasper analysis and mass spectrometry yielded two TFs, ERG and FLI1 shared among the three assays (Fig. 4a). A further five TFs, CREB1 [28], MYB1, SP3, YY1, YY2 with putative binding sites in E2.2 that were detected by mass spectrometry analysis, Jaspar analysis and/or had been previously linked to MEIS1 expression were selected for further analysis (Supplementary Fig. S5a). To understand the possible role of the abovementioned seven TFs in regulating *MEIS1* expression, we targeted the genomic locus of each TF via CRISPR/Cas9. At least two gRNAs per TF were designed and cloned into Cherry colored lentiviral CRISPR-Cas9 vector to reduce/knockout these proteins in MEIS1-GFP-tagged U937 cells (Supplementary Table S3). With the exception of the *MYB* locus, the other targeted TF loci showed ~50% or more editing efficiency in the bulk cells for at least one gRNA with the highest mutation frequency observed at the *FLI1* and *ERG* loci (Supplementary Table S3). Reduction in GFP levels was only observed for FLI1 targeting but not for any other TFs including its family member, ERG (Fig. 4b and Supplementary Table S3 and Supplementary Fig. S5b, c). The concordant reduction in MEIS1 and FLI1 levels were confirmed by immunoblotting (Fig. 4c). The antibody we used for detecting FLI1 also recognizes ERG, which shares the same C terminal sequence as FLI1 [29]. However, only the targeting of FLI1 but not ERG changed the protein level (ERG + FLI1 band) on the western blot suggesting that ERG levels are much lower than FLI1 (Fig. 4c). We further confirmed this observation via qRT-PCR showing much higher *FLI1* mRNA levels compared to *ERG* in U937 cells (Supplementary Fig. S5d).

### FLI1 binds to the E2 enhancer of *MEIS1* and mouse Meis1 binds to the *Fli1* locus

**Fig. 5:**
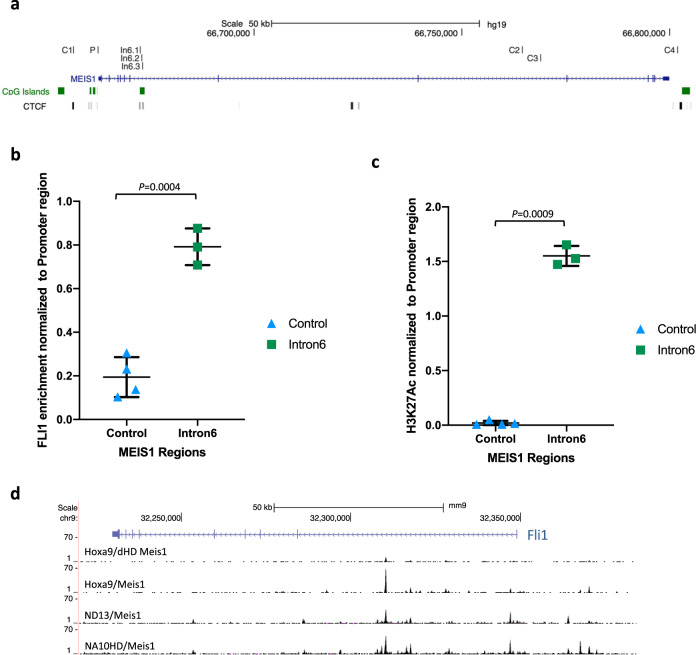
FLI1 regulates *MEIS1* transcription via its association with E2.2 region within intron 6. **a** HG19 UCSC genome browser depiction of the 3 sites of interest surrounding the E2.2 region and four control regions scattered along the *MEIS1* locus in U937 cells. **b** FLI1 binding to the E2.2 region compared to control regions measured by ChIP. **c** Level of H3K27Ac, the active enhancer histone mark, at the E2.2 region compared to control regions measured by ChIP. Welch’s two-tailed *t*-test was used to calculate *p* values. **d** Depiction of Meis1 binding sites within the mouse *Fli1* locus in NUP98-HOXD13/Meis1, NUP98-HOXA10HD/Meis1, Hoxa9/Meis1 versus Hoxa9/deltaHD-Meis1 cells measured by ChIP-seq displayed on the mm9 UCSC genome browser.

To assess FLI1 binding to the E2 enhancer, we performed chromatin immunoprecipitation using FLI1 antibody in U937 cells followed by quantitative PCR to detect FLI1 enrichment at three sites surrounding the E2.2 region compared to four control regions scattered along the *MEIS1* locus (Fig. 5a, b). We detected a significant (*p* = 0.0004) binding of FLI1 to the E2.2 region as well as to the promoter (Fig. 5b). Additionally, H3K27Ac, the active enhancer histone mark, levels were significantly higher (*p* = 0.0009) at the E2.2 region of enhancer E2 compared to control regions (Fig. 5c). We further validated our findings by analyzing publicly available FLI1 and H3K27ac ChIP-seq data for KG-1 (MEIS1-high) and ME-1 (MEIS1-low/medium) human AML cell lines (Supplementary Fig. S6a, b). We detected high FLI1 enrichment in the KG-1 cell line but a much smaller peak in ME-1 cells which also correlates with H3K27ac levels in these lines (Supplementary Fig. S6a). Using ENCODE and BLUEPRINT, enrichment of H3K27ac at the E2 enhancer region of *MEIS1* was further validated in MEIS1-high primary human CD34^+^ cells as well as primary MLL-AF9 AML patients samples compared to MEIS1-low PBMCs and inv [16] respectively (Supplementary Fig. S7a, b).

Given a previous study indicating MEIS1 upregulation of FLI1 in normal hematopoiesis [29], we hypothesised that a positive feedback loop may exist between FLI1 and MEIS1 in AML. Since MEIS1 levels are frequently elevated in normal karyotype AML (CN-AML), we used the murine Hoxa9/Meis1 AML model as a surrogate for CN-AML and performed Meis1 ChIP-seq analysis where we overexpressed HA-tagged wildtype Meis1 or an HA-tagged DNA binding mutant Meis1 (deltaHD-Meis1) with Hoxa9 [21, 30]. We looked for Meis1 binding sites enriched in Hoxa9/Meis1 that were absent or less enriched for deltaHD-Meis1 binding in Hoxa9/deltaHD-Meis1. We detected direct Meis1 binding in the first intron of the mouse *Fli1* gene in Hoxa9/Meis1 which was markedly diminished in the Hoxa9/deltaHD-Meis1 (Fig. 5d). We further validated Meis1 binding to the mouse *Fli1* locus via ChIP-seq in two independent leukemia models, derived from lineage depleted bone marrow cells retrovirally transduced with NUP98-HOXD13/Meis1 (ND13/Meis1) and NUP98-HOXA10 homeodomain/Meis1 (NA10HD/Meis1). All three Meis1 leukemia models show a strong Meis1 peak at the same position of the *Fli1* locus (Fig. 5d).

### FLI1 expression correlates with worse overall survival in AML

**Fig. 6:**
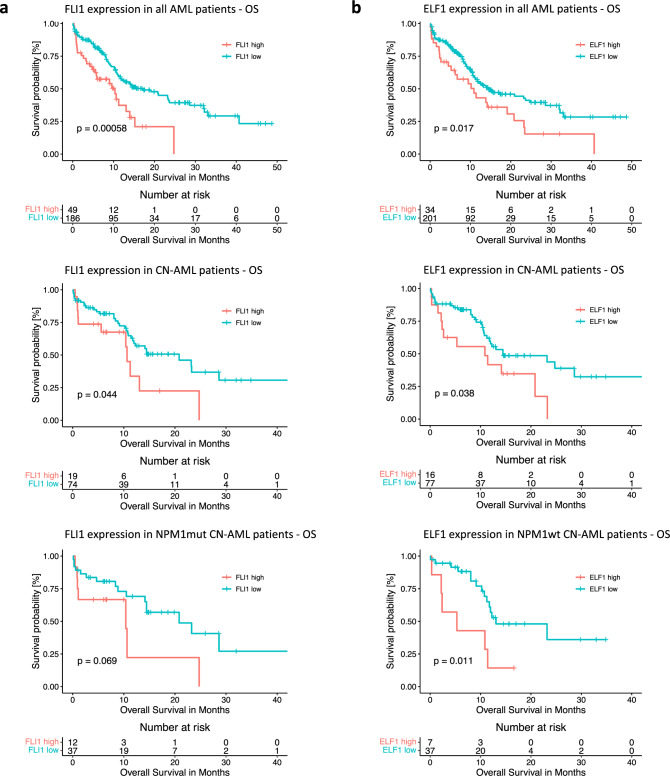
High *FLI1* and *ELF1* transcript levels correlate with worse overall survival in AML. Kaplan-Meier curves depicting overall survival in months based on (**a**) *FLI1* and (**b**) *ELF1* transcript levels calculated in the adult AML subset of the Beat AML cohort. Patients were stratified by gene expression and cutoff for high and low expression groups was calculated using maximally selected rank statistics. *P* values were calculated using log-rank test.

Increased FLI1 protein levels have been previously linked to FLT3 and NPM1 mutated AML and poor outcome [31]. To further understand the relationship between FLI1 and clinical outcome, we analyzed the Beat AML Master Trial dataset [22]. High FLI1 levels correlated with adverse overall survival (OS) in all AML patients (excluding t[15;17]) (*p* = 0.00058) and CN-AML (*p* = 0.044) (Fig. 6a). Additionally, we observed a trend towards worse prognosis with high FLI1 in *NPM1*-mutated CN-AML (*p* = 0.069). We observed a similar correlation for another ETS factor, ELF1, which we had previously shown to bind and upregulate MEIS1 expression [32]. High ELF1 levels correlate with worse overall survival in all AML patients (excluding t(15;17)) (*p* = 0.017), CN-AML (*p* = 0.038), *NPM1*-wildtype CN-AML (*p* = 0.011) and *NPM1*-mutated/*FLT3*-wildtype CN-AML (*p* = 0.046) (Fig. 6b). These correlations further reflect the relationship of MEIS1 expression and patient outcome in all AML patients (excluding t(15;17)) (*p* = 0.0039), CN-AML (*p* = 0.0036), *NPM1*-wildtype CN-AML (*p* = 0.00046) *NPM1*-mutated CN-AML (*p* = 0.072) and *NPM1*-mutated/*FLT3*-wildtype CN-AML (*p* = 0.017) (Supplementary Fig. S8).

## Discussion

HOX proteins and their cofactor MEIS1 are often deregulated in AML especially in normal karyotype [4] and MLL translocation subtypes [3]. However, the mechanisms by which MEIS1 is deregulated in various AML subtypes is yet unknown. Our previous observations show that in addition to the distal and proximal promoter regions, a region within the intron 6 of *MEIS1* displays high DNase I hypersensitivity and histone acetylation levels in U937 cells [12]. Here we present a comprehensive functional analysis of the locus-wide regulatory regions of the human *MEIS1* gene in the U937 AML cell line.

Through the establishment of an endogenous locus reporter system for quick readout of the expression of TFs via flow cytometric analysis, we validate the function of the putative enhancers of the *MEIS1* locus by CRISPR-Cas9 mediated genome editing. Using this pan-locus genome editing approach we identified regions whose loss resulted in the dramatic reduction of MEIS1 expression. Unlike the traditional plasmid-based enhancer assay, such editing occurred at the GFP-tagged endogenous *MEIS1* locus in an AML cell line where the epigenetic landscape was preserved allowing a physiologically relevant readout of the regulatory region modifications. Chromatin capture and insertion/deletion analyses allowed us to pinpoint the region with the greatest impact on *MEIS1* transcription. Our data provides important new functional evidence of the regulatory role of the previously identified candidate enhancer regions for *MEIS1* transcription, and demonstrates the power of CRISPR-Cas9 mediated editing to deduce gene regulation mechanisms via transcriptionally coupled reporter genes. A combination of sequence prediction and oligo pull-down followed by mass-spectrometry detected TF candidates associated with the region of interest. Currently the CRISPR-Cas9 genome editing methods have been used in screening regulatory elements [33, 34]. However, there is a gap between the identification of the enhancer sites and the TFs associated with them [35]. Our approach allowed us to use CRISPR/Cas9-mediated targeting of candidate TFs coupled with the rapid reporter gene flow cytometry readout to identify FLI1 as a key regulator of *MEIS1* in U937 cells. CRISPR-Cas9 mediated mutation of the *FLI1* locus, led to reduced FLI1 levels and a drastic reduction in MEIS1 expression. This finding is in concordance with the observation of high FLI1 protein levels in NPM1 mutated and FLT3-ITD normal karyotype AMLs where MEIS1 levels are also elevated [31]. Interestingly, MEIS1 has been shown to induce hematopoietic progenitor formation in a human pluripotent stem cell system through the upregulation of FLI1 [36]. *MEIS1* deletion in hPSCs downregulated FLI1 and arrested megakaryocytic differentiation whereas the overexpression of FLI1 reversed the impaired megakaryopoiesis caused by *MEIS1* deletion [36]. These findings as well as our data showing direct binding of Meis1 to the *Fli1* locus in mouse, point to a potential positive feedback loop between MEIS1 and FLI1 in normal and aberrant human hematopoiesis.

FLI1, ERG and ELF1 belong to the ETS family of TFs which are frequently involved in tumorigenic translocations [37, 38]. Both FLI1 and ERG have also been implicated in normal hematopoiesis and leukemia formation [39–41]. Given the low expression of ERG in U937 cells, we hypothesize that FLI1 is the key TF regulating *MEIS1* transcription in these cells. A recent study has shown that mouse *Fli1* promotes chromatin looping between the *Meis1* enhancers and promotor in murine erythroleukemia cells [42]. Consistently, our 3 C and Hi-C assays show that mutation of the E2.2 region containing the *FLI1* site reduces promoter-enhancer interaction and chromatin accessibility pointing to the importance of ETS factor binding at the enhancer in addition to the promoter region. Additionally, we have previously shown that another conserved ETS family member, ELF1, positively regulates *MEIS1* transcription in K562 cells via promoter binding [32]. Based on our analysis of the Beat AML dataset, we observe a positive correlation between adverse overall survival and high *FLI1* and *ELF1* transcript levels in all non-acute promyelocytic leukemia patients, especially in CN-AML where MEIS1 levels are high. A recent report also shows a positive correlation between MEIS1 and ELF1 levels in glioma patients and the upregulation of MEIS1 through ELF1 binding to its promoter sequence [43]. These studies and our current findings combined indicate a potential overlapping or compensatory role for ETS factors in the regulation of *MEIS1*. From a therapeutic perspective, it has been shown that mithramycin (plicamycin) inhibits the binding of ERG and FLI1 to DNA in the context of RUNX2, providing a potential new therapeutic avenue for ETS TF driven leukemias [44]. Mithramycin has been tested in a Phase I/II clinical trial for EWS-FLI1 positive tumors as a single agent [45] and is currently in a clinical trial for solid tumors (Clinicaltrails.gov Identifier: NCT02859415).

In summary, we have developed a rapid flow cytometry-based readout for the fine dissection and characterization of the cis-regulatory elements and associated TFs critical for *MEIS1* transcription via CRISPR-Cas9 genetic manipulation. Such a method can be expanded toward the detailed study of other enhancer candidate regions in a pan-genomic approach through gRNA libraries. Our study revealed FLI1 as the candidate key regulator of *MEIS1* expression and a positive correlation between *FLI1* mRNA levels and worse overall survival in MEIS1-high AML subgroups.

## Supplementary information

Supplementary MM and Figures

Supplementary Table S1

Supplementary Table S2

Supplementary Table S3

## Data Availability

All data generated or analysed during this study are included in this published article (and its Supplementary Data file)
