# Supplementary material for: Elucidating the importance and regulation of key enhancers for human MEIS1 expression
Source: Leukemia. 2022 May 27;36(8):1980–9. doi: 10.1038/s41375-022-01602-4 (PMC9343249; doi:10.1038/s41375-022-01602-4)
Supplement: Supplementary file 1 — Supplementary MM and Figures [file 41375_2022_1602_MOESM1_ESM.pdf]

Supplementary Methods and Material

Primers for real-time PCR

	TaqMan set number (IDT)	Region of gene detected
HNRNPL	HS.PT.56a.2939902	ex5-7
MEIS1	HS.PT.51.57188	ex6-7
GAPDH	HS.PT.39a.22214836	ex2-3
MEIS1	Custom design:<br>Probe: 5'-/56-FAM/AGTAGGCAT/ZEN/CCCCTCCACGATGTAT/3IABkFQ/-3'<br>F: 5'-CCTACCCATACGATGTTCCAG-3’<br>R: 5'-ATGGACCTGGCTGCATG-3’	HA-ex2

Primers for 3C assay

GFP_MEIS1_promoter_BsrGI_F	GAGCAAAGACCCCAACGAGA
MEIS1_promoter_BsrGI_probe	/56-FAM/TCGTCCATG/ZEN/CCGAGAGTGATCC/3IABkFQ/

Errc3_region1_F	CCTTCCATGTCTACCCCATAAG
Errc3_region2_F	CACTCGGCAAAAGACCACTA
Errc3_BsrGI_probe	/56-FAM/CCCTCTCAC/ZEN/CATCTACCAACACAGC/3IABkFQ/
#1_66509382F	CCAGTGTCATTTCCCCTCTCC
#2_66521386F	GCCCTCAGCTTTGCTTTGAG
#3_66528557F	GGAAGTCCCCAGTGCAAGTT
#4_66532088F	TGAAATTGACACAGCCGTCA
#5_66536973F	CACCAGAGTCGCTTAGCTGT
#6_66538668F	ACCTCCCCAAAGAGAAAGCC

Primers for ChIP assay

Primer set name	Primer_F	Primer_R
Ctrl_1	CAAATCATACCCCCAACCAC	GCAGCCTTGGTGAATTGTTT
Promoter	TGTGTAAGACGCGACCTGTT	CCGTGCGTGTGTAAAGTGTG
Intron6_1	TCACCCAAGTTTCAGCTGGT	CTCCAGACCGGAGAACTGG

Intron6_2	TACCTGTTTTCCTCGGAGGG	TGGATTTCCGCCGACTTAGC
Intron6_3	TTAAGACACCAGTAGAAGCTGTAATCC	GAGCCCGGAACAGCCAAG
Ctrl_2	AGGCATGATGCAGTGAATTG	CAGGCTAATTCAGGGGTGAT
Ctrl_3	TCTTAGCCAGTCCCAACACC	GTGCAGTCGCAGAACATAGC
Ctrl_4	GCGCTACAAATGGGGAATTA	CCATTGAGCGAGTGCAATAA

Generation and selection of tagged U937 cell lines

This donor template consists of a 1961bp 5’ arm (chr2:66,661,028-66,662,988, hg19) and a 1255bp 3’ arm (chr2:66,662,992-66,664,246, hg19) between which the GFP-P2A-HA sequence was inserted. Both the CRISPR-Cas9 vectors targeting the *MEIS1* translation start site and the donor plasmid were transfected into the U937 cells using the Neon system according to the manufacturer suggested procedure (Thermo Fishier Scientific, Waltham, MA, USA). 48 hours after transfection, individual GFP^+ cells were sorted and further analyzed after 1 week. The mono-allelic and bi-allelic tagged clones were confirmed by PCR analysis based on the detection of both wildtype (WT) *MEIS1* alleles and *MEIS1* alleles bearing the GFP-P2A-HA insert at the same time.

Lentivirus production and transduction

Lentivirus supernatant were collected at 36 hr and 60 hr post transfection, filtered and stored in -80°C until usage. For transduction, 100 µl crude viral supernatant was added to 5x10^{4} cells seeded in 24 well plates in 400 µl RPMI++ supplemented with 4 µg/ml protamine sulfate (MilliporeSigma, St. Louis, Missouri, USA). Cells were transduced overnight, washed and expanded afterwards.

PCR amplification and sequencing of the gRNA targeted region

To check the efficiency of genome editing using gRNA 10 (E2.1) or 11 (E2.2) in the MEIS1 tagged allele, 2 rounds of PCR reactions were done. 1st round of PCR used a forward primer 5'-CGACCACTACCAGCAGAACA-3' that is inside the tagged sequence within the GFP fragment, and a reverse primer 5'-CCGCTGTTTGTGTCCACTTT-3' that is within the intron 6 region of *MEIS1*. 1/100 of 1st round PCR product was used as the template for 2nd round PCR with the target-specific primer set (either 10 or 11).

Tandem Mass Spectrometry

The biotin labeled WT oligo for DNA pull down was 5'-ATACTAGGCGGTATCCCGGAGGGCTAAGTC- 3'. In order to decrease the nonspecific binding, 10-fold of non-biotin labeled mutant oligo (5' -

ATACTAGGCGGTAT*ATTAA*AGGGCTAAGTC- 3’) was added prior to the addition of the labeled WT oligo. Proteins were eluted from the beads, extracted in 0.1% trifluoroacetic acid plus 50% acetonitrile, dried, and analyzed by a Dionex UltiMate 3000 liquid chromatography separation system interfaced with a QSTAR XL tandem mass spectrometer (Applied Biosystems, Foster City, CA, USA). Peptides were identified using the Analyst (Applied Biosystems, Foster City, CA, USA) and MASCOT6 software. Results are presented as observed isotopic mass which was calculated from the mass-to-charge ratio (m/z) for each peptide and compared with the predicted mass.

Hi-C assay and analysis

Hi-C was performed on Parental (MEIS1-GFP-tagged Cherry$^+$GFP$^+$) and E2.2 CRISPR’d U937 (MEIS1-GFP-tagged Cherry$^+$GFP$^-$) cells as previously described (1). The restriction enzyme HindIII was used for chromatin digestion.

Reads generated from Hi-C libraries were aligned by Hi-C User Pipeline HiCUP (https://www.bioinformatics.babraham.ac.uk/projects/hicup/) and used to identify a filtered set of interactions pairs (2). Normalized interaction matrices were generated using HOMER (http://homer.ucsd.edu/homer/) by comparison against background models at 1 Mb and 10kb resolutions(3), and visualized using HiCPlotter (4). Principle component analysis was used to extract PC1, differentiating regions of active and inactive chromatin.

Similarities in accessibility between treatments were identified by correlating these PC1 values.

Transcription Factor binding analysis to E2.2 FLI1 gRNA locus

The probability of a human transcription factor binding to the E2.2 FLI1 gRNA locus was assessed using the R package TFBStools (5). DNA sequences of the E2.2 gRNA locus, along with base pairs taken 5 base pairs up and downstream, were used as input for transcription factor binding analysis. Binding motifs for human transcription factors were extracted from the JASPAR 2020 database (6) and then converted into position weight matrices using TFBStools. To determine which transcription factors were binding to the E2.2 locus, a % sequence match was utilized as a threshold and results were then filtered based on the provided probability scores.

Chromatin immunoprecipitation (ChIP) assay in U937 cells

Rabbit Anti-ERG/FLI1 monoclonal antibody (ab92513, Abcam, Cambridge, United Kingdom) which recognizes both ERG and FLI1 was used. Four regions within the *MEIS1* locus were selected as internal controls. Real-time PCR was performed in the StepOne Real-Time PCR System (Applied Biosystems, Foster City, CA, USA). All data

are presented as the ratio of the PCR readings of a given primer set over one of the internal control regions, versus the PCR readings of the input control.

MEIS1 expression in AML cell lines

Log2 Transcript Per Million (TPM) normalized reads were retrieved from the Cancer Cell line Encyclopedia (7). The results were filtered for cell lines originating from patients with Acute Myeloid Leukemia, from which MEIS1 expression was extracted and then plotted.

ChIP-seq of Histone modifications and FLI1 binding at the E2 enhancer of MEIS1

FLI1 and H3K27ac ChIP-seq tracks in AML cell lines were taken from ascension series GSE158794 (8). Bigwig files for histone ChIP-seq of PBMCs and $CD34^+$ cells were downloaded from the ENCODE portal (9) (https://www.encodeproject.org) with the following identifiers: ENCFF501OWH, ENCFF537GCQ, ENCFF961HTB, ENCFF804VSQ, ENCFF915EPW, ENCFF379MJL, ENCFF391QSF, ENCFF623URK, ENCFF704EGV, ENCFF093GGJ. Histone ChIP-seq tracks for AML patients (MLL-AF9 and inv(16) were downloaded from Blueprint Epigenome (www.blueprint-epigenome.eu) from the International Human Epigenome Consortium data portal (10).

References

1. Belton JM, McCord RP, Gibcus JH, Naumova N, Zhan Y, Dekker J. Hi-C: a comprehensive technique to capture the conformation of genomes. Methods. 2012;58(3):268-76.
2. Wingett S, Ewels P, Furlan-Magaril M, Nagano T, Schoenfelder S, Fraser P, et al. HiCUP: pipeline for mapping and processing Hi-C data. F1000Res. 2015;4:1310.
3. Heinz S, Texari L, Hayes MGB, Urbanowski M, Chang MW, Givarkes N, et al. Transcription Elongation Can Affect Genome 3D Structure. Cell. 2018;174(6):1522-36 e22.
4. Akdemir KC, Chin L. HiCPlotter integrates genomic data with interaction matrices. Genome Biol. 2015;16:198.
5. Tan G, Lenhard B. TFBSTools: an R/bioconductor package for transcription factor binding site analysis. Bioinformatics. 2016;32(10):1555-6.
6. Fornes O, Castro-Mondragon JA, Khan A, van der Lee R, Zhang X, Richmond PA, et al. JASPAR 2020: update of the open-access database of transcription factor binding profiles. Nucleic Acids Res. 2020;48(D1):D87-D92.
7. Ghandi M, Huang FW, Jane-Valbuena J, Kryukov GV, Lo CC, McDonald ER, 3rd, et al. Next-generation characterization of the Cancer Cell Line Encyclopedia. Nature. 2019;569(7757):503-8.
8. Thoms JAI, Truong P, Subramanian S, Knezevic K, Harvey G, Huang Y, et al. Disruption of a GATA2-TAL1-ERG regulatory circuit promotes erythroid transition in healthy and leukemic stem cells. Blood. 2021;138(16):1441-55.
9. Davis CA, Hitz BC, Sloan CA, Chan ET, Davidson JM, Gabdank I, et al. The Encyclopedia of DNA elements (ENCODE): data portal update. Nucleic Acids Res. 2018;46(D1):D794-D801.
10. Yi G, Wierenga ATJ, Petraglia F, Narang P, Janssen-Megens EM, Mandoli A, et al. Chromatin-Based Classification of Genetically Heterogeneous AMLs into Two Distinct Subtypes with Diverse Stemness Phenotypes. Cell Rep. 2019;26(4):1059-69 e6.

Supplementary Figure S1

a

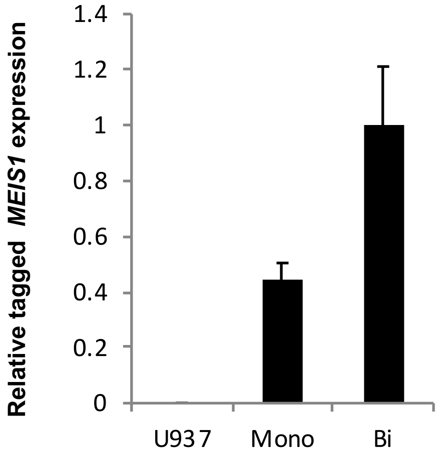

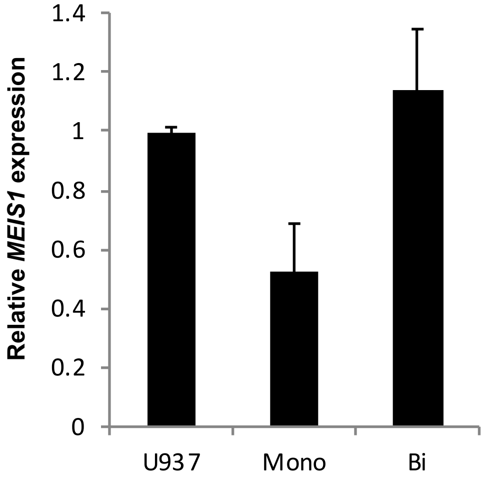

b

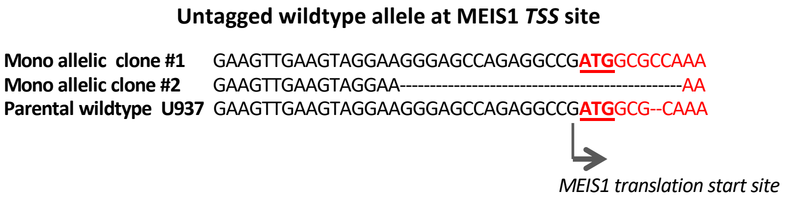

Supplementary Figure S1. Characterization of monoallelic and biallelic MEIS1-tagged clones. (**a**) HA-tagged *MEIS1* mRNA levels measured by primers spanning the tag region to exon 2 of *MEIS1* (left panel) and the amount of total *MEIS1* transcript measured by real time PCR (right panel). (**b**) Abrogation of MEIS1 expression in mono-allelic tagged clones due to a frame shift mutation.

Supplementary Figure S2

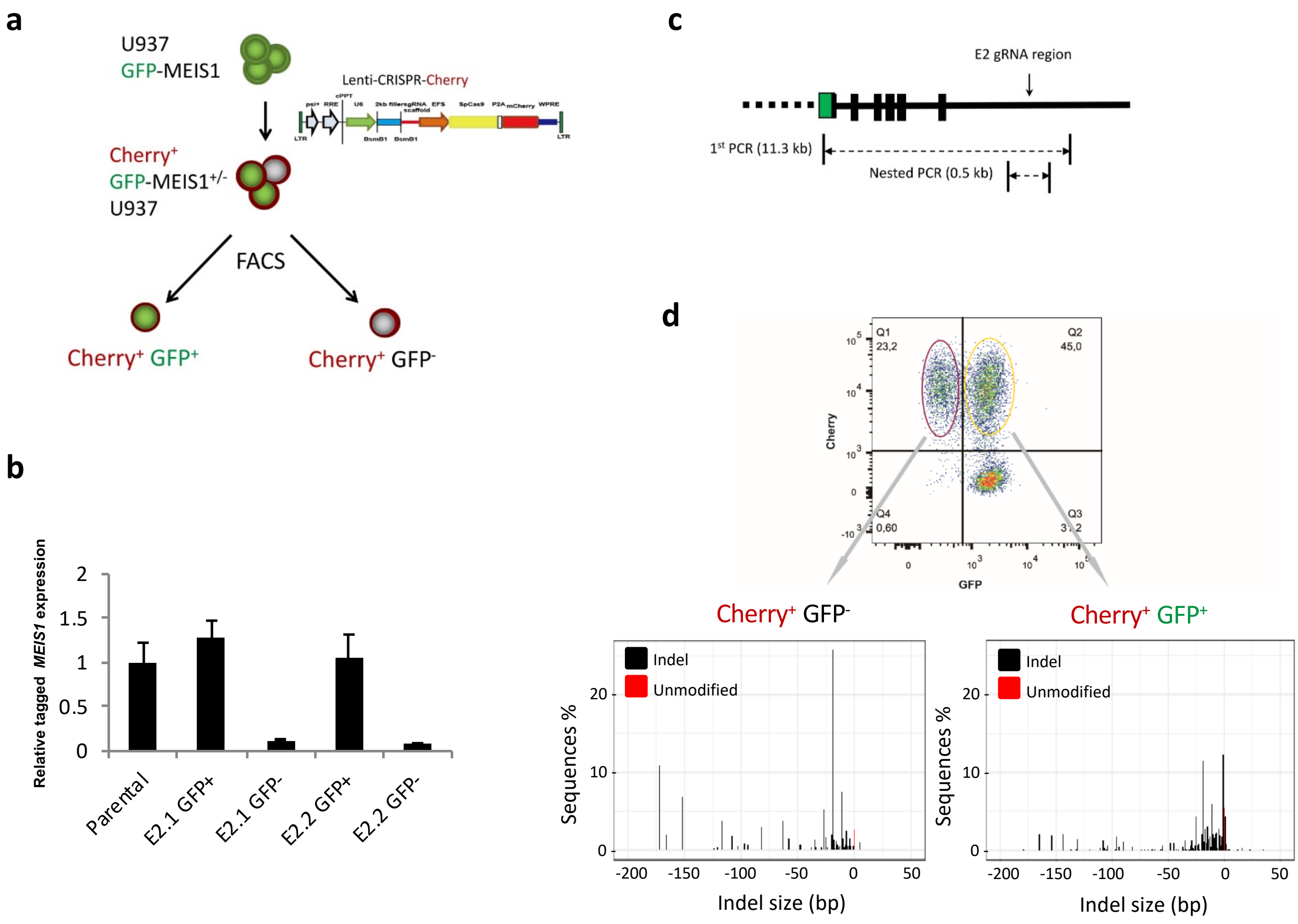

Supplementary Figure S2. Characterizing the E2.1 and E2.2 targeted mono-allelic MEIS1-GFP-tagged cells. (**a**) The Cherry colored lentiviral CRISPR-Cas9 mediated genome editing vector system. (**b**) *MEIS1* mRNA expression of Cherry$^+$GFP$^+$ cells and Cherry$^+$GFP$^-$ cells from E2.1 and E2.2 targeted cells measured by real time PCR. (**c**) PCR amplifying the E2 region in MEIS1-GFP-tagged Cherry$^+$GFP$^+$ cells and Cherry$^+$GFP$^-$ cells. (**d**) Targeted site of the E2.1 region in MEIS1-GFP-tagged Cherry$^+$GFP$^+$ cells and Cherry$^+$GFP$^-$ cells detected by sequencing.

Supplementary Figure S3

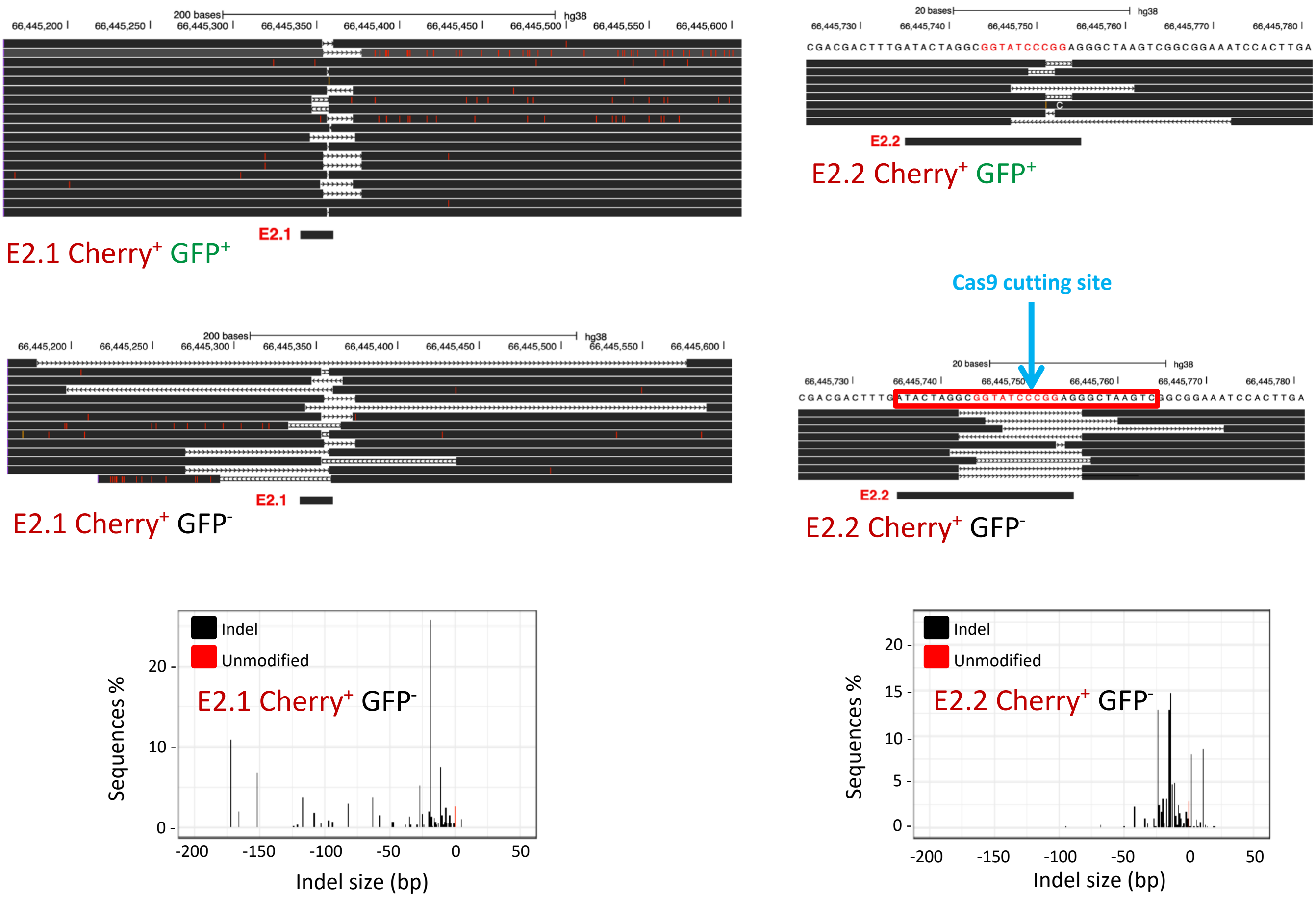

Supplementary Figure S3. E2.1 and E2.2 induce varied indel size distribution. Comparison of the genomic region targeted by E2.1 and E2.2 in MEIS1-GFP-tagged Cherry⁺GFP⁺ cells and Cherry⁺GFP⁻ cells via PCR amplification and sequencing. FLI1/ERG binding site sequence is shown in red font and the sequence of the pull-down oligo is indicated in red box.

Supplementary Figure S4

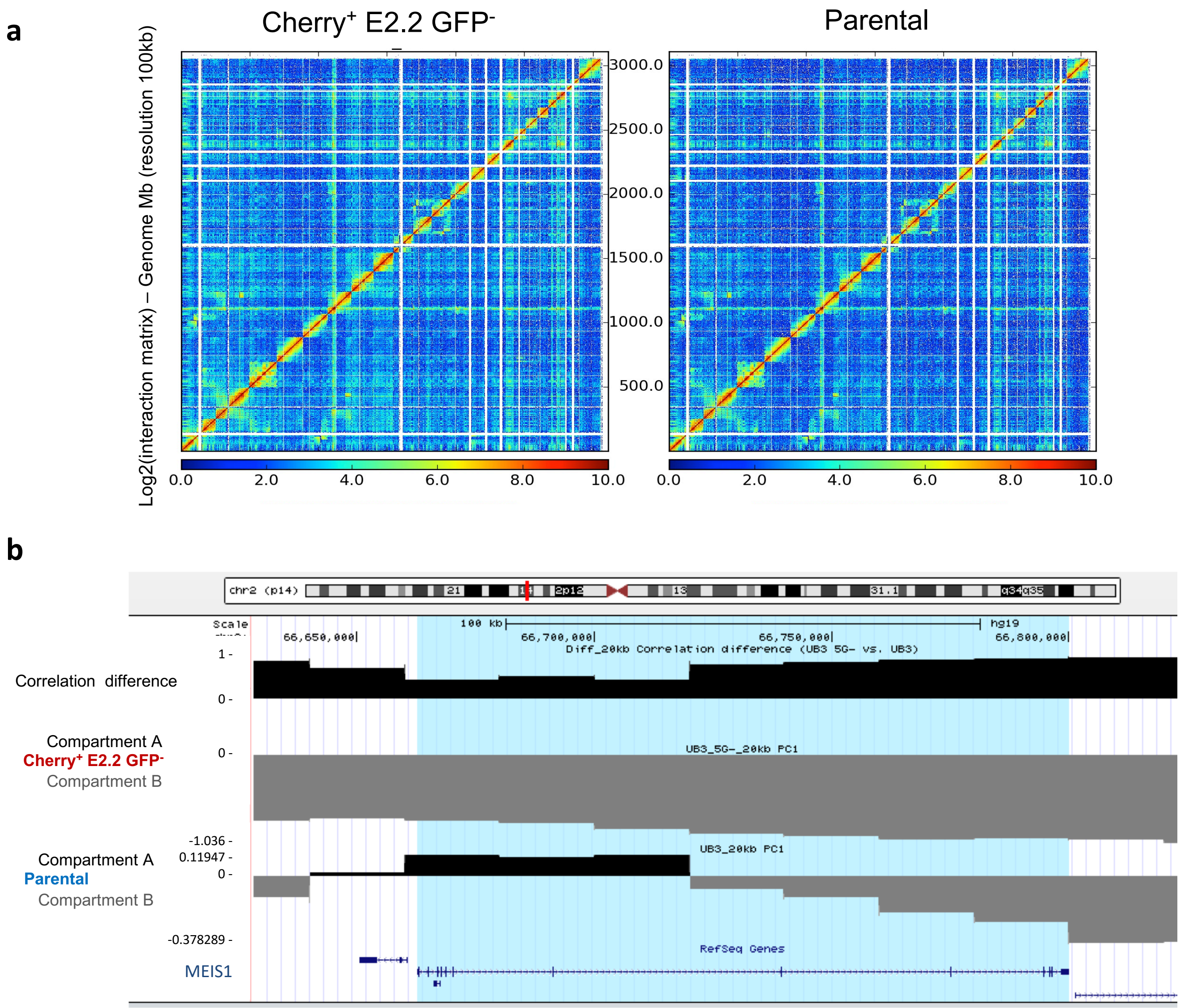

Supplementary Figure S4. Differential compartmentalization of the genome observed by Hi-C analysis in Parental (MEIS1-GFP-tagged Cherry$^+$GFP$^+$) and E2.2 CRISPR'd U937 (MEIS1-GFP-tagged Cherry$^+$GFP$^-$) cells. (**a**) Whole genome interaction map at 1Mb resolution. (**b**) Visualization of the partitioning of the MEIS1 locus at 10kb resolution. Chromatin compartments, determined by principal component analysis (PCA) showing PC1 for the Parental (MEIS1-GFP-tagged Cherry$^+$GFP$^+$) and E2.2 CRISPR'd U937 (MEIS1-GFP-tagged Cherry$^+$GFP$^-$) cells that depicts compartment A (closed chromatin shown in black) from compartment B (open chromatin shown in grey). The correlation difference shows a shift to closed chromatin (compartment B) in the E2.2 CRISPR'd U937 (MEIS1-GFP-tagged Cherry$^+$GFP$^-$) cells.

Supplementary Figure S5

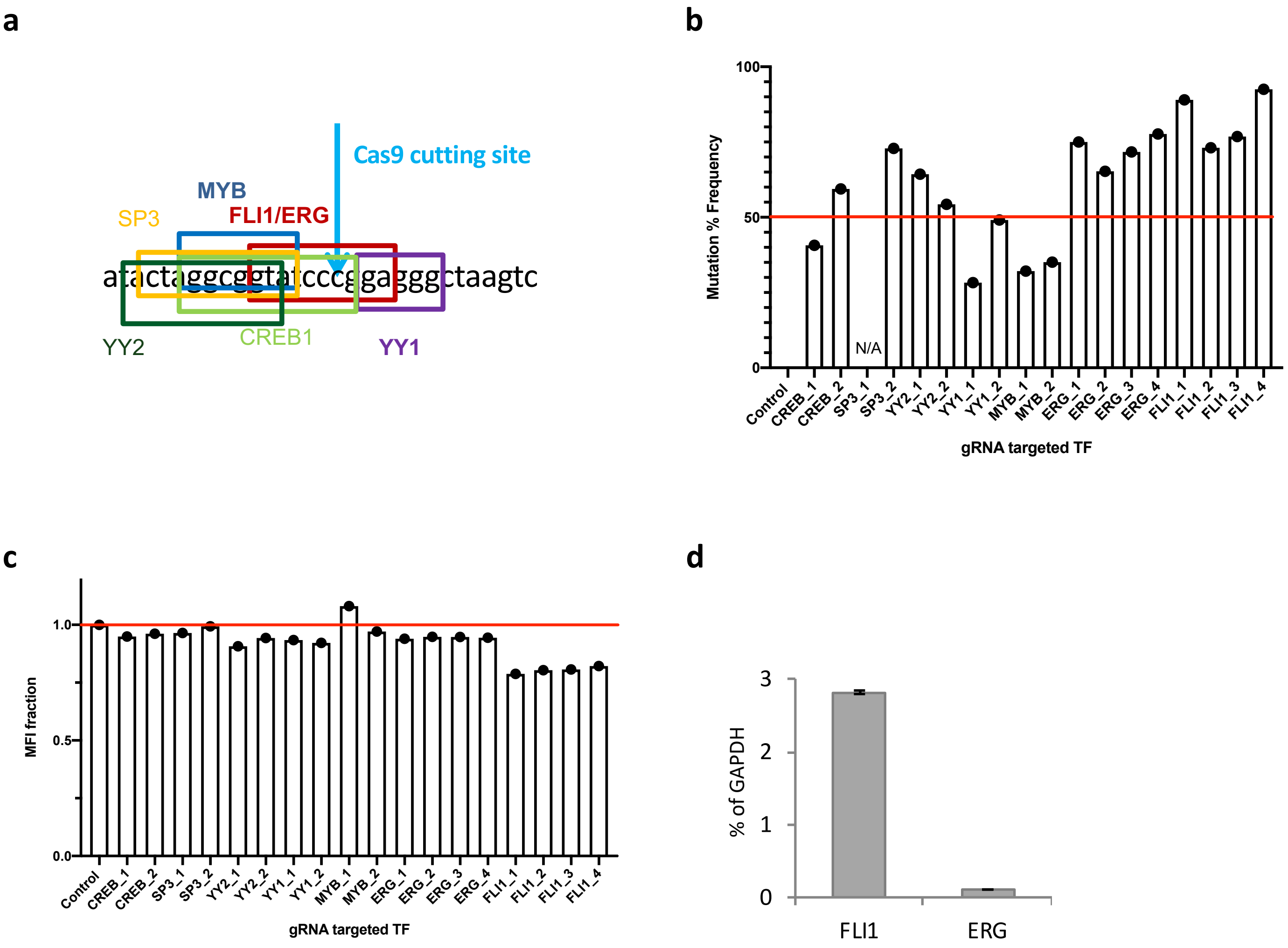

Supplementary Figure S5. FLI1 but not ERG regulates *MEIS1* transcription. (**a**) Putative E2.2 binding transcription factors identified via Jasper analysis and mass-spectrometry. (**b**) Editing efficiency of CRISPR-Cas9 targeting transcription factors in MEIS1-GFP-tagged U937 cells measured by MiSeq. (**c**) GFP levels of MEIS1-GFP-tagged U937 cells with transcription factors targeted by CRISPR-Cas9. (**d**) mRNA levels of *FLI1* and *ERG* in MEIS1-GFP-tagged cells measured by qRT-PCR. MFI: mean fluorescence intensity.

Supplementary Figure S6

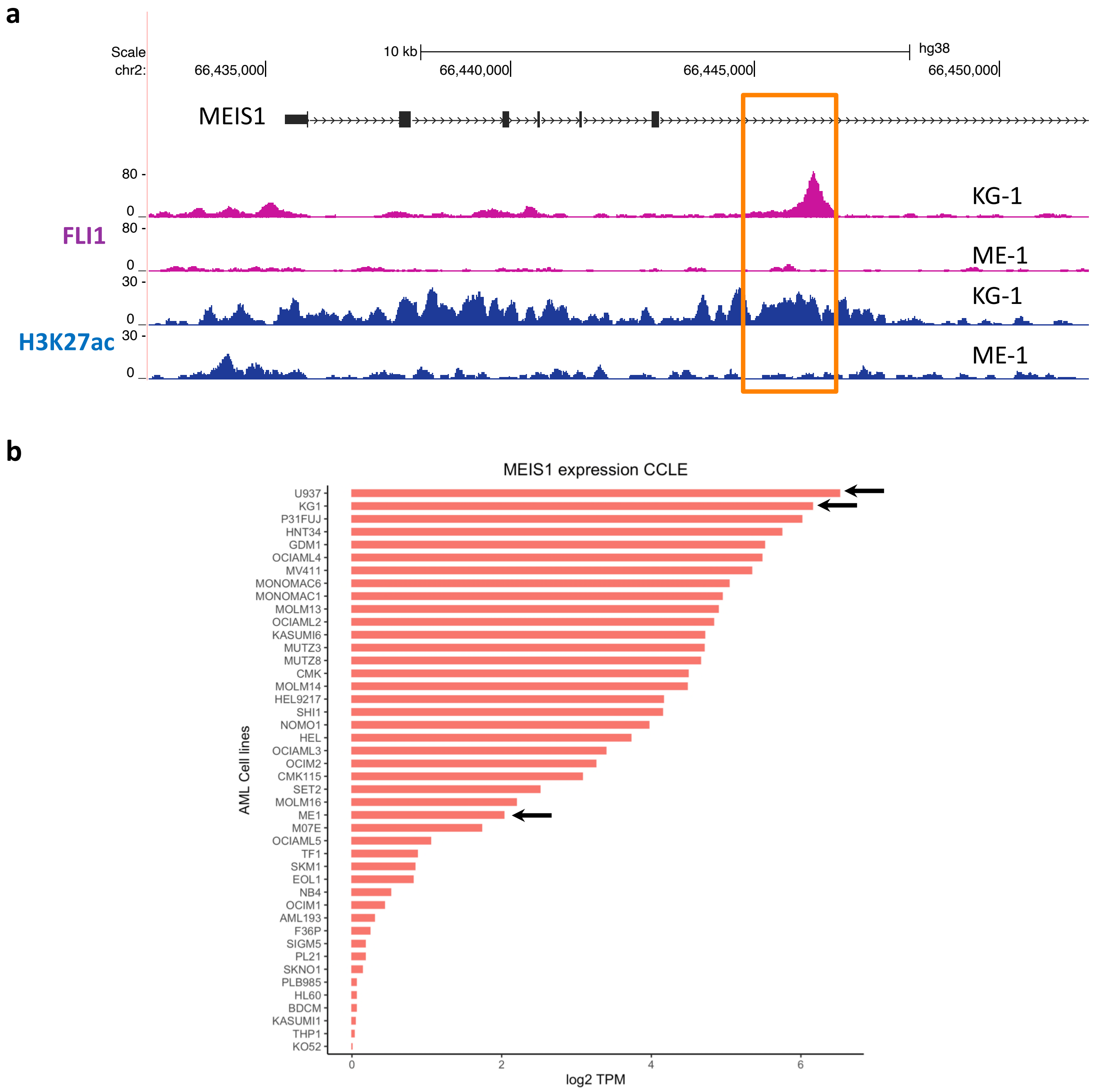

Supplementary Figure S6. UCSC genome browser shot of FLI1 binding and H3K27ac at the E2 enhancer of MEIS1 in human leukemia cell lines. (**a**) FLI1 binding (pink) to the MEIS1 E2.2 region and H3K27ac (blue) in MEIS1 high KG1 cells compared to the MEIS1 low/medium ME-1 cell line. (**b**) MEIS1 transcript levels in various human leukemia cell lines depicted from the Cancer Cell Line Encyclopedia (CCLE). Black arrows indicate U937, KG-1 and ME-1 cell lines.

Supplementary Figure S7

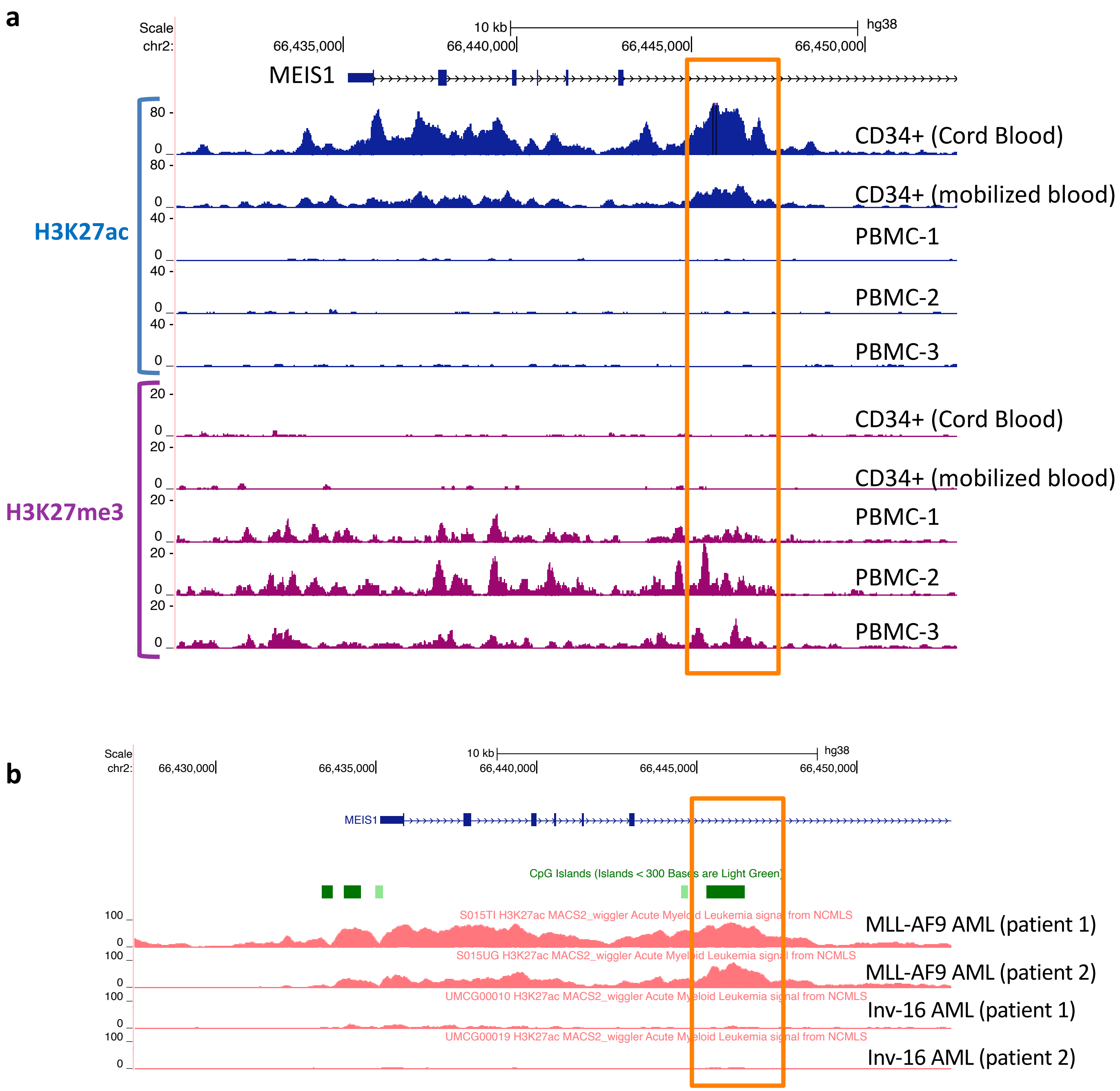

Supplementary Figure S7. UCSC genome browser shot of H3K27ac and me3 of the MEIS1 locus in primary hematopoietic human cells. (**a**) H3K27ac (blue) and H3K27me3 (purple) tracks from the human ENCODE roadmap-epigenomics for two primary CD34+ and three peripheral blood mononuclear cells (PBMCs) samples aligned to Hg38 human genome. (**b**) H3K27ac tracks from BLUEPRINT Epigenome in two MEIS1 high (MLL-AF9) and two MEIS1 low (Inv-16) primary AML patients. The orange boxes delineates the E2 region of MEIS1.

Supplementary Figure S8

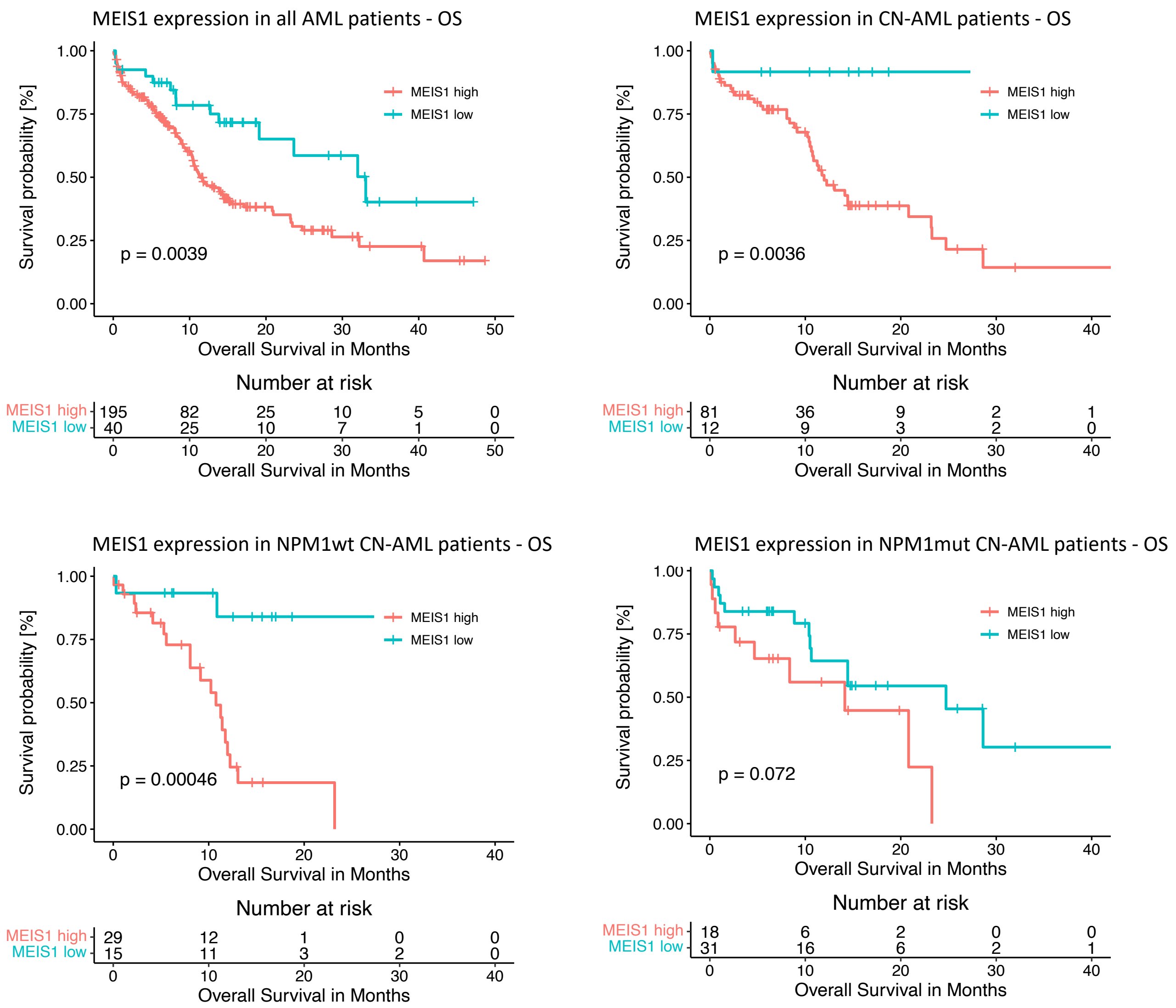

Supplementary Figure S8. High MEIS1 transcript levels correlate with worse overall survival in AML. Kaplan-Meier curves depicting overall survival in months based on *MEIS1* transcript levels calculated in the adult AML subset of the Beat AML cohort. Patients were stratified by gene expression and cutoff for high and low expression groups was calculated using maximally selected rank statistics. P-values were calculated using log-rank test.
