# Supplementary material for: Elucidating the importance and regulation of key enhancers for human MEIS1 expression
Source: Leukemia. 2022 May 27;36(8):1980–9. doi: 10.1038/s41375-022-01602-4 (PMC9343249; doi:10.1038/s41375-022-01602-4)
Supplement: Supplementary file 2 — Supplementary Table S1 [file 41375_2022_1602_MOESM2_ESM.pdf]

gRNA #	gRNA sequence	%GFP- Cherry + (bi allelic)	%GFP- Cherry + (mono allelic)	mutation frequency %
1	CAGGCGATTTATGCGAGAAA	0	2.12	45.60
2	GCCTGTTAATCAATCGAGTT	0.28	5.57	69.74
3	TTTACTTTGCGCTTAAGAGT	0.59	6.41	78.95
4	ACGGCTGCCTACCGAGATTT	1.04	6.53	96.81
5	TCATTTTCCAGTCGACCGCA	0.86	6.09	91.77
6	GGTTTCGCCGAGCCGGGTTG	5.91	21.5	96.01
7	GTGTAAGACGCGACCTGTTA	0	0.97	34.41
8	TTATGGCCACCACTACTTCC	1.11	9.15	84.03
9	GGCTAATGAACCGTACAGAC	2.31	14.4	80.47
10	ACAGTGCCACGGCCGCCGGT	8.05	37.2	95.25
11	ATACTAGGCGGTATCCCGGA	6.92	28.9	88.96
12	TCCCAGTCTTCCGGGCCGCC	0	0.6	17.34
13	GAGCCGAGAGCGCGGCGGTC	1.85	21.1	89.84
14	GAGAACGATGCGGGTTCGAC	0.59	11.4	87.41
15	CGCCCTCCTCCGAAGAAGCG	2.78	20.2	87.91
16	ACTCGTTCAGGAGGAACCCC	1.88	15.4	85.74
17	TGATTTCTGTGCATAAACGG	0.07	2.45	63.47
18	CTTTTCTAGTCGCAATTAAT	0	0	5.72
19	CTGTGAGTGCCAGTGCGTAT	0.055	2.56	78.07
20	ACCATGTTTAACTCCCCGGT	0.17	7.13	91.03
21	CTTACCAATTGTTCACTTGA	0.04	2.57	19.41
22	TGAGGACACTTATCCGAAAG	0	1.17	0.00
23	GGGGATTTCGAAAAACGACT	0.1	0.44	63.01
ctrl AAVS1	GGGGCCACTAGGGACAGGAT			
