# Supplementary material for: Elucidating the importance and regulation of key enhancers for human MEIS1 expression
Source: Leukemia. 2022 May 27;36(8):1980–9. doi: 10.1038/s41375-022-01602-4 (PMC9343249; doi:10.1038/s41375-022-01602-4)
Supplement: Supplementary file 3 — Supplementary Table S2 [file 41375_2022_1602_MOESM3_ESM.pdf]

Jaspar /Wt_MassSpec/Mut	Wt_MassSpec/Mut_Competed	Jaspar/Wt_MasSpec	Jaspar	Mut_Competed	Wt_MassSpec
**ERG**	RPS11	**SP3**	REL	STK10	IFI16
**FLI1**	ACTB		HIC2	EIF1AY	RPL23
	YBX1		RHOXF1	NSD1	RALYL
	**YY1**		MEIS1	DSP	GNL3
	CDCA7L		KLF11	SOX4	DNAJB8
	KRT19		GSC	DNAJB1	HSPB1
	H1-3		DMRTC2	RPS5	PTPRR
	H4C9		TBX4	PCDHA1	AIFM1
	H2AZ2		ZIC5	KRT72	H3-3A
	TMA16		GATA3	ANP32E	RALGAPA2
	RPS2		KLF2	DNAJB4	SRCAP
	H2BC12		ELF5	UAP1L1	PGAM5
	RTEL1		CENPB	RPS29	CTSB
	SEPTIN7		NEUROD2	MT1L	PDCD11
	PTMA		NKX2-8	PSIP1	PNKP
	H2BC18		GSX2	NPIPB2	RABL3
	RPL26		NKX2-3	USF3	PHB2
	H2AC7		NFIX	MT1A	RBMX
	RPS18		TBX5	PM20D2	SUB1
	KRT14		FOXP3	NSD3	KIF20B
	ACTG2		ELF4	IQGAP1	C5
	HSPA7		MGA	SAP18	RPS27L
	TUBA4A		HOXC8	CEP170B	EIF5B
	EWSR1		DMRT3	EIF1AX	NSMCE2
	TOP1MT		HMBOX1	SFI1	MAX
	DAZAP1		NFKB2	DSG1	EIF3B
	H1-5		ZKSCAN1	RPS24	RPN1
	H2AC21		TEAD3	KRT71	TFAM
	KRT24		HOXC10	RPL37AP8	HLA-B

	CHCHD3		SPDEF	AGPS	RPL7A
	KRT9		TFAP2C	RIN1	VIM
	MATR3		KLF5		DES
	POTEI		ETV6		NONO
	ATP5F1EP2		TBX6		CHRNA3
	DDX5		GSC2		SRSF7
	HNRNPD		MEIS3		HECTD1
	HNRNPA0		HOXB3		SFPQ
	ATP5F1E		E2F1		CANX
	SMARCA5		THAP1		TUBAL3
	H2BC9		EVX1		EBNA1BP2
	H2AZ1		ATOH7		BAZ1B
	HNRNPCL4		TBX3		FUBP3
	TUBB2A		ETS2		MAD1L1
	GTF2I		SP9		SMC3
	KRT77		ETS1		RALY
	LMNB1		BARHL1		PTBP3
	SCAF1		HOXB6		DDX27
	KRT76		TFAP2B		SDF4
	KRT27		TBX15		ATXN7
	SRP14		IRF6		HNRNPK
	TUBA1A		STAT1		IKZF1
	KIF4A		NR2C2		RRP1
	RPS7		MZF1		SMARCA2
	HMGN1		EVX2		RRS1
	KRT73		OTX1		SNRPB2
	CHMP2A		HOXA9		SMCHD1
	TUBB2B		TFAP2E		SLC25A5
	LYZ		DMRTA2		ATP5F1A
	H2AC12		TFAP2A		**CREB1**
	HNRNPA3		RUNX2		HDGF

	RPL37A		KLF6		RPL3L
	H2AC14		SRY		PRPF19
	HSPA2				CCAR2
	RPS6				ZNF326
	KRT8				RPL12
	H2BU1				NA
	CHERP				CCDC137
	HMGN2				SMARCA4
	POTEJ				H3-4
	SP100				TENM3
	HSPA6				VDAC1
	KRT1				CSE1L
	LMNB2				PHB
	H2BC13				HLA-C
	CTSG				ITGB2
	KRT25				ERLIN2
	HNRNPA2B1				FUS
	KRT36				KRT84
	PDIA3				H3C14
	SNRNP70				GATAD2B
	RPL11				TUFM
	KRT10				NSUN5
	H1-0				RPL6
	TUBB4B				DNAJB6
	HNRNPCL2				SPTBN1
	H2BC1				SSBP1
	RPL8				RPS3
	HSPA1L				RPL3
	KRT4				STMN2
	KIF2C				PPT1
	POTEF				SUPT16H

	RNASE3					TREX1
	H2AC6					GATAD2A
	H2AW					ABCA12
	H2AC13					TBL2
	KRT28					ATAD3B
	H1-6					MRPS31
	TUBA8					HNRNPL
	RREB1					PLEC
	KRT13					TEX11
	KRT35					ATF1
	TUBB8					TAF11
	KRT5					RPS16
	HDGFL2					SRSF3
	THYN1					CDKN2AIP
	NEFH					FBXL19
	ACTA1					ATAD3A
	H2AC18					RPL27A
	RPS4Y2					KHSRP
	KRT6C					TARDBP
	H2BC21					TRIM28
	RPL15					POLB
	RPL31					PTBP1
	APOH					STMN1
	HSPA1A					RAN
	HNRNPUL1					VDAC2
	HNRNPDL					ERLIN1
	FLG2					FUBP1
	KRT6B					ATP5F1C
	RPS25					PRPH
	TOP2B					KRT7
	HSPD1					RPL13

	RPS3A				PRIM2
	H2AC1				DNAJB2
	DEK				H3C1
	ACTA2				CEP295
	HSPA5				RPS27
	EMD				MAP2K6
	KRT26				DDX17
	ALB				
	TUBB				
	CLEC2B				
	H1-1				
	ACTBL2				
	TUBB6				
	RPL17				
	H1-10				
	HNRNPR				
	KRT74				
	H2BC14				
	UBTF				
	TUBB8B				
	LMNA				
	KRT38				
	RPL34				
	UHRF2				
	KRT12				
	HNRNPM				
	RPL28				
	H2AC8				
	UHRF1				
	RPL18A				
	NPM1				

	TCERG1				
	KRT16				
	RRP15				
	HNRNPCL1				
	RSL1D1				
	TUBB3				
	NOP2				
	HNRNPAB				
	SINHCAF				
	TOP2A				
	SYNCRIP				
	RPS4X				
	KRT33B				
	CXXC1				
	TUBA3C				
	PTMS				
	KRT31				
	RPS4Y1				
	TUBA1C				
	H2AX				
	RPS14				
	KRT79				
	RPL30				
	ACTC1				
	ACTG1				
	POTEE				
	H2BC3				
	HNRNPU				
	MPG				
	LOC102724334				
	H2BC15				

	H2BC5				
	KRT3				
	KRT37				
	RPL26L1				
	KRT78				
	NCL				
	RBM28				
	**YY2**				
	RPL14				
	KRT6A				
	HNRNPA1				
	KIF2A				
	KRT18				
	SMC1A				
	HSPA8				
	TCOF1				
	H2AC20				
	ALDH3B2				
	IMMT				
	RBMS2				
	RPS19				
	HP1BP3				
	SRP9				
	H2BC11				
	TUBB4A				
	RPL9				
	FBL				
	TUBB1				
	H1-4				
	TUBA3E				
	MNDA				

	RBMS1				
	RPS8				
	KRT15				
	TUBA1B				
	KRT75				
	BANF1				
	HNRNPC				
	H1-2				
	KRT17				
	RPL4				
	ZMYM3				
	SURF6				
	KRT32				
	PPAN				
	WDR76				
	H2BC17				
	HNRNPUL2				
	PARP1				
	TOP1				
	ARID3B				
	KRT2				
	**MYB**				
	HRNR				
	DCD				
	H2AJ				
